# Computerized cognitive remediation therapy effects on resting state brain activity and cognition in schizophrenia

**DOI:** 10.1038/s41598-017-04829-9

**Published:** 2017-07-06

**Authors:** Fengmei Fan, Yizhuang Zou, Yunlong Tan, L. Elliot Hong, Shuping Tan

**Affiliations:** 1 Beijing Huilongguan Hospital, Peking University, Beijing, 100096 P.R. China; 2 State Key Laboratory of Cognitive Neuroscience and Learning & International Data Group/McGovern Institute for Brain Research, Center for Collaboration and Innovation in Brain and Learning Sciences, Beijing Normal University, Beijing, 100875 China; 3 Maryland Psychiatric Research Center, Department of Psychiatry, University of Maryland School of Medicine, Baltimore, USA

## Abstract

This study aimed to test how an 8-week training using computerized cognitive remediation therapy (CCRT) would modify resting brain functional activity and improve cognitive function in patients with schizophrenia. Twenty-seven patients with schizophrenia were recruited and randomized into two groups: CCRT or treatment-as-usual (TAU). The CCRT group received 40 sessions of computerized cognitive training over an eight-week period. There was a significant treatment group × time interaction on the processing speed (trail making test: F = 8.14, *P* = 0.01) and a trend in problem solving (mazes test: *P* = 0.06). Post-hoc tests showed that CCRT but not TAU significantly improved scores from baseline to end-of-treatment on these two cognitive assessments. For the resting brain functional activity, significant group × time interaction effect was found in the medial prefrontal cortex (mPFC)/anterior cingulate cortex (ACC) and brainstem pons region. Post-hoc tests showed that there was significant increased activity in the mPFC/ACC in CCRT but not TAU group. In this small sample study, computerized cognitive remediation therapy is shown to enhance mPFC/ACC activity even at resting state and improve cognitive function in patients with schizophrenia. If replicated, this community and clinic accessible therapy may assist cognitive remediation effort for people with schizophrenia.

## Introduction

Cognitive deficits are core features of schizophrenia, contribute substantially to poor functional outcome of the patients^1, 2^, and are likely as important as positive and negative symptoms for clinical treatment of the illness^3^. Many forms of cognitive remediation therapy (CRT) have been developed to aid treatment for cognitive deficits in the general population and in schizophrenia. Computer-assisted training for cognition remediation has received increased attention due to its accessibility. As in most cognitive remediation, computer-assisted training aims to improve attention, memory, language and/or problem-solving areas. An emerging body of research has shown that computerized cognitive remediation training (CCRT) produced a significant improvement in neurocognitive function^4–8^, and the effects of CCRT on cognition were durable^9–12^. CCRT also has been shown to improve neurocognitive function that can be generalized to untrained neurocognitive domains and may also impact symptoms and work functioning in patients with schizophrenia^6, 7, 13, 14^.

Several studies focused on exploring cognitive training induced cerebral changes in healthy populations using magnetic resonance imaging (MRI) techniques. After 2 weeks of memory training, increased activations were found in nine healthy male adults in the right inferior frontal gyrus and the right intraparietal sulcus during a visual spatial working memory task using functional magnetic resonance imaging (fMRI), with the activation values decreased at the time of consolidation of performance gains after 4 weeks^15^. Four-week working memory training in healthy university students alters resting-state brain activity and connectivity^16^, indicated that intrinsic brain can be affected by cognitive training. Thicker cortex and decreased cortical activation were observed after three months of a visual-spatial problem-solving computer game in adolescent girls^17^. Cognitive training could also mitigate the age-related functional alterations using resting fMRI, thereby helping older adults maintain brain health^18^. In a review on practice-induced structural and functional changes in the brain in older adults, Degen and colleagues demonstrated that practice is associated with volumetric increases using structural MRI, reorganization of neural network recruitment with changes in functional activity levels^19^. The effects of cognitive rehabilitation on resting brain activity and connectivity have been investigated in Parkinson’s disease^20, 21^ and multiple sclerosis^22^. In schizophrenia, cognitive enhancement therapy not only protects against gray matter loss^23, 24^. Additionally, one fMRI study found that patients with schizophrenia who received successful CRT had significantly improved brain activation in regions associated with working memory, particularly the frontal cortical areas, where improvement was defined as the differences by a fMRI memory task compared with baseline resting conditions^25^. However, the different activities under such fMRI design could be due to changes of neuronal activity at either the task state or the resting state. In the current design, we tested the hypothesis that CRT effects on cognition and brain activity may occur at neuronal activity during the resting state irrespective of performance during fMRI tasks. To our knowledge, whether the activation in resting fMRI is changed in patients with schizophrenia who received CRT has not been investigated.

Resting activity can be assessed using amplitude of low-frequency fluctuation (ALFF), which detects the regional intensity of spontaneous fluctuations in the blood-oxygen-level dependent (BOLD) signal of the brain^26^. ALFF has been used in resting fMRI studies in schizophrenia. In a meta analysis, ALFF abnormalities were detected in a number of regions implicated in the disorder including reduced activity in medial orbitofrontal cortex, superior temporal gyrus, sensorimotor, and posterior parietal cortices^27^. ALFF abnormality has also been found in other psychiatric populations. For example, attention deficit hyperactivity disorder (ADHD) has been associated with reduced ALFF in inferior frontal cortex^26^. Many of these implicated areas are closely related to cognitive performance such as processing speed^28, 29^, working momory^30^, executive function or problem solving^31^. Therefore, we tested the hypothesis that successful CCRT would enhance ALFF in cortical regions associated with reduced ALFF in patients and/or key cognitive functions.

The aim of the current study is to detect neurocognitive function changes and ALFF changes after CCRT in symptomatic but clinically stable patients with schizophrenia. Previous CRT showed that skills with the most consistent improvement by such CRT paradigms are executive-function, attention, and memory^32, 33^. Therefore, we further test the relationships between ALFF changes and changes in these neurocognitive performances.

## Materials and Methods

### Study Design

In Beijing Huilongguan Hospital, we randomized patients with schizophrenia in a single-blind, randomized controlled trial comparing the effect of computerized cognitive remediation therapy (CCRT) and versus standard therapy without CCRT. The study was approved by the Ethics Committee of the Beijing Huilongguan Hospital, and all experiments were performed in accordance with relevant guidelines and regulations. After a detailed explanation of the study protocols, all participants and their guardians gave written informed consent for participation in this study and the publication of the results.

### Participants

**Figure 1:**
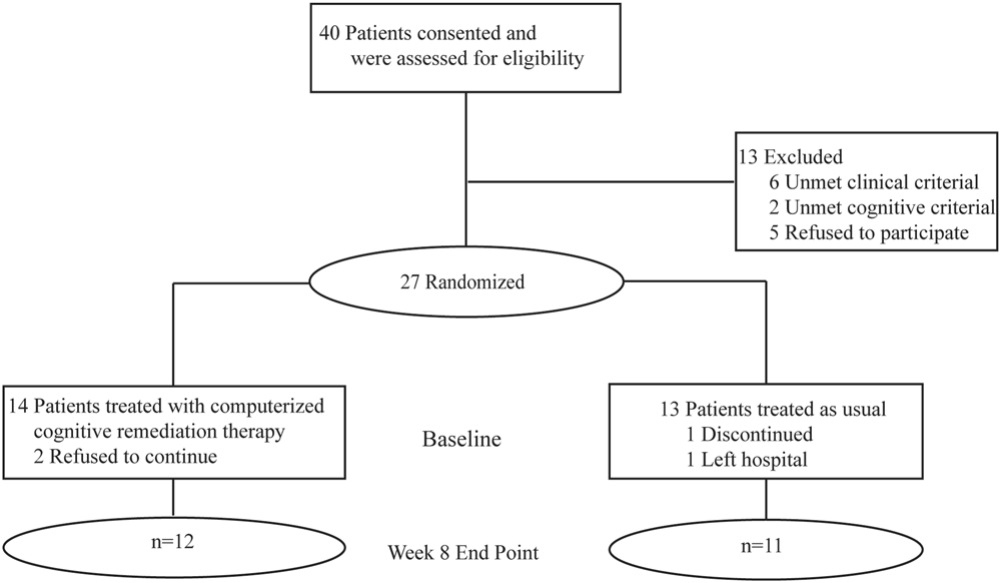
Consort Diagram.

Twenty-seven voluntarily admitted inpatient patients with schizophrenia (16 males and 11 females, mean age 39.7 ± 7.2 years, ranged from 24 to 50) were recruited from Beijing Huilongguan Hospital. They had chronic schizophrenia and were symptomatic that required prolonged hospitalizations but were clinically stable during the study period. Inclusion criteria included: (1) DSM-IV diagnostic criteria for schizophrenia; (2) In stable clinical state according to their psychiatrist; (3) No less than 6 years of education; (4) Right-handed confirmed by the short version of the Edinburgh Handedness Scale. All patients who completed the study remained hospitalized during the duration of the trial. Exclusion criteria included: (1) A history of head trauma; (2) Concurrent or previous substance dependence or alcoholism; (3) Gross brain organic disease confirmed T2 MRI; (4) Tardive dyskinesia; (5) Learning disability or mental retardation. Twenty-three patients met these criteria and were randomized and four dropped out before randomization for various reasons (see Fig. 1). Demographic data are given in Table 1.

The patients were randomly assigned to a CCRT group or to a treatment-as-usual (TAU) control group, stratified by age, gender and education to match these demographic parameters between groups. Randomization was independently conducted by a psychiatrist not otherwise involved in the study at the completion of all baseline assessments. A random number table was used to generate lots that were drawn for sealed envelopes, which assigned the participants to CCRT or TAU group.

### Study Outcomes

Psychiatric symptomatology was evaluated using the Positive and Negative Syndrome Scale (PANSS) with validated Chinese language version^34, 35^. The neurocognitive assessments were examined using trail making test (Part A for processing speed), mazes test (for problem solving), spatial span test and digit sequencing test (for working memory), selected from the MATRICS Consensus Cognitive Battery (MCCB) with validated Chinese versions^36–38^. All the raw scores were transformed into a T score with a mean of 50 and an SD of 10 according to Chinese norms^36, 38^, higher scores of T-value represent better cognitive performance. Clinical assessment, neurocognitive functions and resting state fMRI were assessed in a two-day period immediately before CCRT and then in a two-day period immediately after the last CCRT session. TAU patients were assessed using the same schedule. The clinical assessment rating (Positive and Negative Syndrome Scale, PANSS) was conducted by two attending psychiatrists, who were trained to be reliable and had achieved intraclass correlation coefficient (ICC) value of 0.88 before the trial. Two clinical psychologists, who had at least 5 years experience in psychometric testing, examined the neurocognitive functions. Before our study started, the two raters had achieved reliability at ICC values 0.80 or above on all tests before the trial. All the raters were blind to group assignment.

### Interventions: Cognitive Remediation Therapy

The CCRT group received 40 sessions of computerized cognitive training over an eight-week period. It included five 45-minute sessions each week for 8 weeks, making a total of 40 sessions.

The treatment procedure consisted of participants training on computer software programs with guidance/assistance from a therapist, who supervised 4 participants in CCRT group at the same time. Each participant was assigned to a cubicle separated by a panel. We developed a software named computerized cognitive remediation therapy (CCRT) in Chinese, adopting the principals of cognitive remediation therapy mainly from the English literature^8, 11, 39–44^. A CCRT session involved 30 different cognitive training tasks focusing on improving cognitive skills in working memory, cognitive flexibility, planning, and social function especially facial emotion recognition and emotion management. Before the treatment trial, the CCRT task battery was calibrated by testing its feasibility and tolerability in 10 other patients with schizophrenia conducted over a 2 months period. For each CCRT training task, there was a series of difficulty levels graded from easy to hard (changed automatically based on the performance of the patient), so that an errorless learning environment can be provided. The participants in this group received antipsychotic medication treatment plus general psychosocial interventions provided by their psychiatric treatment team independent of the CCRT study.

The participants in TAU group received antipsychotic medication treatment plus general psychosocial interventions such as psychiatric education. All of the interventions received in TAU group were generally used in almost every inpatient in the hospital. Patients on TAU completed all baseline and end-of-study clinical, cognitive, and fMRI assessment.

### fMRI Protocol

MRI was acquired on a Siemens 3 Tesla MRI Trio scanner using a 12-channel headcoil (Erlangen, Germany). Head motions were minimized by snugly fixed using a belt and foam pads, and wore earphone to reduce noise. Structural MRI was obtained with the following parameters for registration purpose. T1-weighted images were acquired covering the whole brain with sagittal 3D-MPRAGE (magnetization prepared rapid acquisition gradient echo) sequence: echo time (TE) = 3.01 ms, inversion time (TI) = 900 ms, repetition time (TR) = 2300 ms, flip angle (FA) = 9°, field of view (FOV) = 256 mm × 256 mm, matrix size = 512 × 256, thickness/gap = 1/0.5 mm. The resting-state functional images were obtained with the following parameters: TR = 2000 ms, TE = 30 ms, FA = 90°, in-plane resolution = 64 × 64, FOV = 210 mm × 210 mm, 30 axial slices, thickness/gap = 4/0 mm and 210 volumes (7 minutes).

### Data Analysis

The first 10 volumes were discarded for fMRI signal to reach a steady state, and for the subjects to get used to the scanner noise. Part of the preprocessing of resting state fMRI data were performed using the Statistical Parametric Mapping (SPM5, http://www.fil.ion.ucl.ac.uk/spm/software/spm5) package, including slice timing, head motion correction (a least squares approach and a 6-parameter spatial transformation) and spatial normalization to the Montreal Neurological Institute (MNI) template (resampling voxel size = 3 mm × 3 mm × 3 mm). During normalization, mean functional volume created after head correction was co-registered to T1 image. T1 image was normalized into MNI template, producing normalization parameters, which were used to write into all functional images. Subjects with head motion more than 4.0 mm of maximal translation (in any direction of x, y or z) or 4.0° of maximal rotation throughout the course of scanning were excluded from further analysis (excluding two subjects, one from each group). Subsequent data preprocessing were performed using software DPARSF (http://restfmri.net/forum/index.php) implemented with MATLAB, including removal of linear trends, band-pass filtered (0.01 < f < 0.08 Hz) to reduce the very low-frequency drift and high-frequency respiratory and cardiac noise^45, 46^, calculation of ALFF, and spatial smoothing (full width at half maximum = 8 mm Gaussian kernel). ALFF at each voxel was taken as following: the time series for each voxel was transformed at the frequency domain and the power spectrum was then obtained. The square root was calculated at each frequency of the power spectrum and the averaged square root was obtained across 0.01–0.08 Hz at each voxel. This averaged square root was taken as the ALFF^26^. The ALFF of each voxel was divided by the individual global mean of ALFF within a brain-mask, which was obtained by removing the tissues outside the brain.

### Statistical analysis

All variables were assessed for normal distribution using Shapiro-Wilk test, and variables found to be not normally distributed were transformed to achieve normal distribution. Effects of CCRT on clinical and cognitive assessments were examined by two-way repeated measure analysis of variance where group (CCRT vs. TAU) were between-subject factor and time (before and after) with within-subject factor, with age, gender and education as covariates. Post-hoc tests were carried out using pairwise comparisons on those scores that showed significant group × time interaction effect using Bonferroni correction for multiple comparisons.

For the resting state fMRI data, paired t tests were performed on ALFF in the CCRT group to obtain brain regions of interest (ROI) showing significant time effect in the CCRT group, considering age, gender and education as covariates. Then, two-way repeated measure analysis of variance was performed on ALFF of these ROIs where group (CCRT vs. TAU) were between-subject factor and time (before and after) with within-subject factor, to identify ROIs that showed significant group × time interaction effect on ALFF.

Statistical thresholds for repeated measure analysis and paired t test on ALFF used voxel *P* < 0.01 and cluster size corresponding to corrected *P* < 0.05 based on Monte Carlo simulation (AlphaSim in AFNI). In order to analyze the relationship between the ALFF changes and the cognitive changes in patients with schizophrenia, we performed Pearson correlation analysis on brain regions showing significant treatment effect.

## Results

### Demographic and clinical characteristics

The demographic and clinical characteristics of the participants are shown in Table 1. At baseline, there were no significant difference between CCRT group and TAU group on age, gender, education and onset age. All participants received second-generation antipsychotic agents. Four patients in CCRT group received two different antipsychotic medications (Clozapine plus Risperidone, Sulpiride plus Quetiapine, Olanzapine plus Quetiapine, Clozapine plus Olanzapine). Another three participants in TAU group received two antipsychotic agents (Clozapine plus Sulpiride, Quetiapine plus Olanzapine, Clozapine plus Risperidone). No significant difference was detected on antipsychotic medication dose between the two groups. During the two months treatment, there was no change of antipsychotic medication type or dose in any individual of the two groups.

**Table 1:** Demographics of participants.

	CCRT group (n = 12)	TAU group (n = 11)	T or χ2	*P*
Gender(M/F)	7/5	5/6	0.04	0.84
Age (years)	39.67 ± 6.21	41.45 ± 3.53	0.84	0.41
Education (years)	12.08 ± 2.15	11.27 ± 1.68	1.00	0.33
Illness duration (years)	16.30 ± 7.41	18.55 ± 5.37	0.80	0.43
Onset age	24.3 ± 4.47	22.36 ± 5.75	0.86	0.40
Risperidone	2	7		
Clozapine	3	3		
Olanzapine	4	2		
Sulpiride	1	1		
Quetiapine	3	1		
Ziprasidone	2	0		
Perphenazine	1	0		
Dose of antipsychotic medication (Chlorpromazine- equivalent mg/day)	553.90 ± 253.57	421.98 ± 189.68	1.402	0.176

Note: TAU = treatment-as-usual; CCRT = computerized cognitive remediation therapy PANSS = Positive and Negative Syndrome Scale.

### Clinical and Cognitive Outcomes

**Figure 2:**
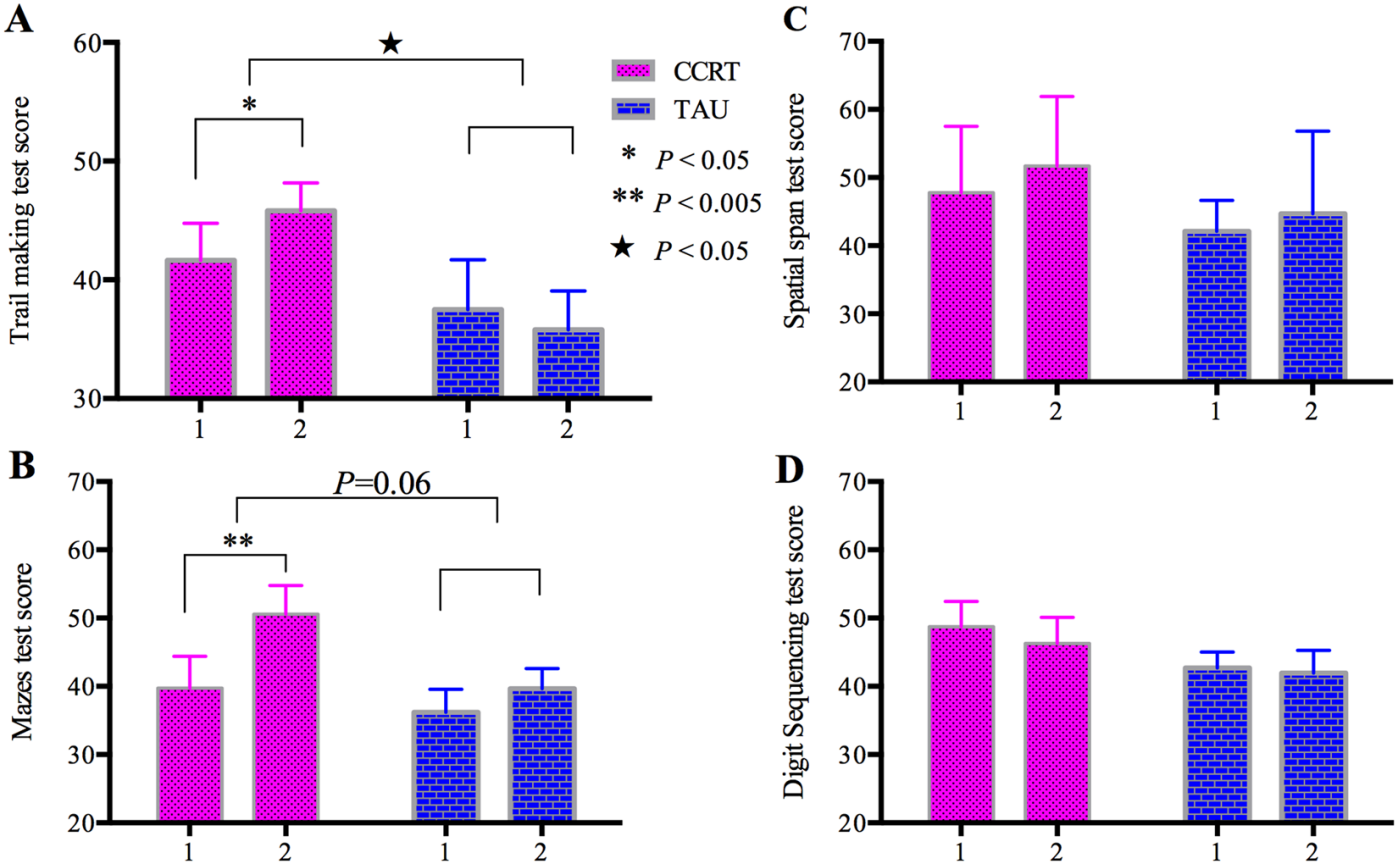
Comparison of cognitive function at baseline and post-treatment in computerized cognitive remediation therapy (CCRT) and treatment as usual (TAU) groups, including trail making test, mazes test, spatial span test and digit sequencing test. Note: in x axis, 1 = baseline, 2 = post-treatment. Scores in y axis represent T-scores of cognitive performance. ^★^Means significant group × time interaction, *means significant treatment effect within group.

The repeated measure analysis of cognitive function showed significant interaction between group and time on trail making test score for processing speed (F_(1,18)_ = 8.14, *P* = 0.01). There was a trend of significant interaction in maze test score for problem solving (F_(1,18)_ = 4.11, *P* = 0.06, see more detail in Fig. 2, Table S1). Post-hoc tests in CCRT group showed significant improvement in trail making (*P* = 0.03, Fig. 2A), and maze test score (*P* = 0.001, Fig. 2B). In TAU group, there was no significant change in trail making (*P* = 0.22) or maze test (*P* = 0.29). The two working memory tasks showed no significant group × time interaction (all *P* > 0.65, Fig. 2C and D, Table S1), and no significant improvement in each group separately.

On clinical symptoms, there was no significant group × time interaction in PANSS total score, PANSS positive, PANSS negative and PANSS general psychosis score within the two groups.

### Resting state fMRI- ALFF changes

**Figure 3:**
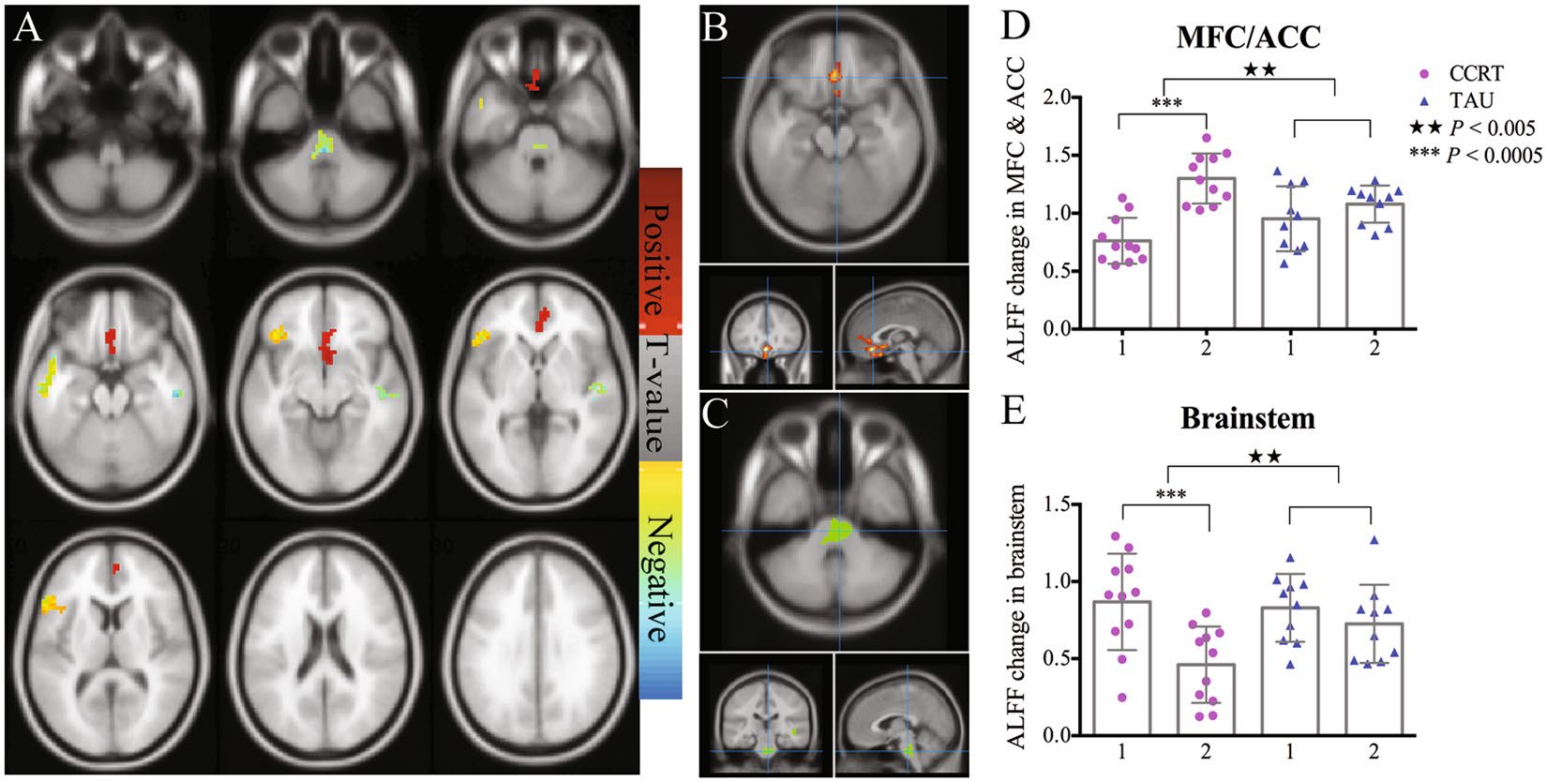
Amplitude of low-frequency fluctuation (ALFF) changes after CCRT. Paired t-test results in CCRT group were shown in Fig. 2A. And two areas shown significant interaction between group and time are plotted in Fig. 2B and C. The scatter plots of the two regions are also drawn (D and E). Note: in x axis of Fig. 2D and E, 1 = baseline, 2 = post-treatment. ^★^Means significant group × time interaction, *means significant treatment effect within group.

Following the statistical analysis set out above, paired t test in CCRT group showed significant increased ALFF after treatment in the medial prefrontal gyrus and anterior cingulate cortex (mPFC/ACC), olfactory cortex and superior temporal gyrus and decreased ALFF in left inferior frontal gyrus, insula, middle temporal gyrus, inferior temporal gyrus, temporal pole and brainstem (Table 2, Fig. 3A, corrected *P* < 0.05). Using these as ROIs, we extracted the ALFF in both CCRT and TAU and performed two-way repeated measure analysis of variance. Two ROIs showed significant group × time interaction: mPFC/ACC (F_(1,18)_ = 17.56, *P* = 0.001, Fig. 3B and D), and brainstem (F_(1,18)_ = 11.07, *P* = 0.005, Fig. 3C and E). Compared with baseline and TAU, CCRT resulted in significantly higher ALFF in mPFC/ACC, and significantly lower ALFF in brainstem (Fig. 3D and E).

**Table 2:** Brain regions showing significant treatment effect of ALFF between baseline and post-treatment in CCRT group.

Brain region	Hemisphere	MNI cordinate of peak voxel [x; y; z]	BA	Cluster size (voxel)	T value	Cohen’s d
Inferior Frontal Gyrus; Insula	L	[−48; 27; 0]	45/47/44	167	−3.51	−0.62
Middle Temporal Gyrus; Inferior Temporal Gyrus; Temporal Pole	L	[−51; −12; −18]	21/38/20	116	−3.51	−0.24
Superior Temporal Gyrus	R	[57; −21; 18]	22/21	66	8.02	−0.32
Medial prefrontal cortex; ACC; Olfactory cortex	—	[0; 30; −15]	11/32/25	225	9.50	1.34
Brainstem	—	[0; −33; −39]	−	76	−3.50	−1.12

### Correlations with clinical and demographic variables

**Figure 4:**
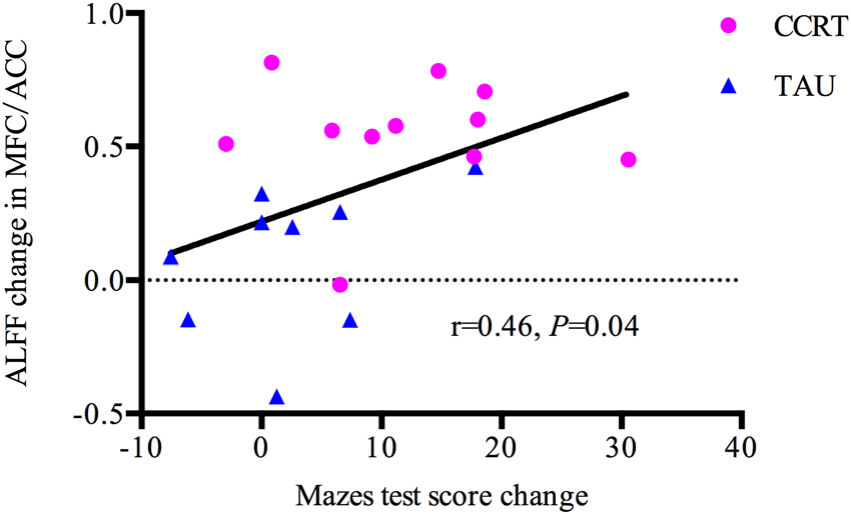
The correlation between improvements of mazes score and mean ALFF changes at baseline and post-treatment in combined CCRT and TAU group.

For brain regions where there were significant repeated-measures group $\times$ time interaction effects, we explored the relationships between ALFF changes and improvement of cognitive score. The results showed that the ALFF changes in mPFC/ACC were significantly correlated with mazes score changes in combined CCRT and TAU group (r = 0.46, *P = *0.04, Fig. 4). Additionally, we also examined the relationship between ALFF and medication dosage (chlorpromazine equivalents) and found that medication dosage was not correlated with ALFF in any of the group. Finally, the brainstem activity did not show significant correlation with cognitive performance changes.

## Discussion

In the current study, we report that neurocognitive function improvements and ALFF changes in resting state fMRI after computerized cognitive remediation therapy. Compared to the baseline and treatment as usual, ALFF were found increased in the medial prefrontal and anterior cingulate cortex area and reduced in the brainstem in CCRT group. Thus, our data suggested that a 2-month CCRT might impact cognition and brain function even in chronic patients with schizophrenia at least in the short term.

The neurocognitive improvement was detected in trail making and mazes scores after CCRT. The finding is in line with previous studies that cognitive remediation training can achieve significant gains in cognitive performance^8, 11, 39–42, 47^. The areas of increased resting activity amplitude in schizophrenia were in medial frontal and anterior cingulate regions, which were consistently associated with abnormal function in schizophrenia^29, 31, 48, 49^. Our finding of increased ALFF in frontal regions is consistent with previous studies showing that similar frontal regions may be dysregulated in the context of working memory task performance^25, 50, 51^. Task-related frontal lobe activation was increased through CRT^25, 52^ or cognitive exercises^53^. Honey and colleagues found similar results with another type of therapy, risperidone, which was associated with improvements in frontal cortex functioning over a shorter period of time^54^. Moreover, the results may also be consistent with the notion that dysregulation of medial frontal regions is associated with self-directed thoughts^48^. This might provide a neurophysiological basis for cognitive improvements^52, 55^.

All the study participants were chronic patients admitted for long-term inpatient treatment and had been on a stable dose of medication for at least 2 months prior to entering the study and dose of antipsychotic medication remained constant throughout the study period including 2 months. Therefore, the results are unlikely due to changes in medication.

Improvement of maze score was significantly correlated with ALFF changes at the mPFC/ACC. However, this significant finding was significant only on combined CCRT and TAU groups, and may reflect overall relationship between cognitive improvement and ALFF change in mPFC/ACC and not necessarily reflect CCRT specific effect. This maze task required subjects to attend to, organize, rehearse, and update task information. Regions related to attention and cognitive control are known to include the medial prefrontal and anterior cingulate regions^48, 56^.

The current study has several limitations. One of these is that participants were all chronic schizophrenia and taken antipsychotic medication. However, cognitive deficits in chronic patients represent a large clinical and economic burden for the patients and their families. Identifying easily accessible ways to improve cognitive function in chronic patients and understand the mechanisms of improvement are critically needed for advancing research and developing more effective treatment. The study designed to examine immediate or short-term effect of cognitive training; it is unclear whether these effects might last, which is another limitation. Also, sample size is small and our finding encourages subsequent, larger scale studies to replicate the finding. Moreover, it’s very important to explore treatment effect across age, age of illness onset and duration of illness in a larger sample in future. Overall, the findings from this study suggested that 2-month computerized cognitive remediation therapy might enhance specific domains of cognitive function in chronic, stable schizophrenia and do so in part by influencing their resting state brain function. If replicated, this community and clinic accessible therapy may assist cognitive remediation effort for people with schizophrenia.

### Data Access and Responsibility

The principal investigator, Shuping Tan, had full access to all of the data in the study and takes responsibility for the integrity of the data and the accuracy of the data analysis.

### Trial registration

The registration number is ChiCTR-TRC-08000249 in Dec 24, 2008.

## Electronic supplementary material

Supplementary information
