# Supplementary material for: Computerized cognitive remediation therapy effects on resting state brain activity and cognition in schizophrenia
Source: Sci Rep. 2017 Jul 6;7:4758. doi: 10.1038/s41598-017-04829-9 (PMC5500543; doi:10.1038/s41598-017-04829-9)
Supplement: Supplementary file 1 — Supplementary information [file 41598_2017_4829_MOESM1_ESM.pdf]

Computerized cognitive remediation therapy effects on resting state brain activity and cognition in schizophrenia

Fengmei Fan[1,2], Yizhuang Zou[*1], Yunlong Tan[1], L. Elliot Hong[3], Shuping Tan[1*]

1 Beijing Huilongguan Hospital, Peking University, Beijing 100096, P.R. China

2 State Key Laboratory of Cognitive Neuroscience and Learning & International Data Group/McGovern Institute for Brain Research, Center for Collaboration and Innovation in Brain and Learning Sciences, Beijing Normal University, Beijing 100875, China

3 Maryland Psychiatric Research Center, Department of Psychiatry, University of Maryland School of Medicine, Baltimore, USA

Corresponding Author:

Shuping Tan, M.D, PHD; Yizhuang Zou, M.D, PHD

Beijing Huilongguan Hospital, Peking University, Beijing 100096, P.R. China.

Email: shupingtan@126.com, yzouy@263.net.

Running title: Cognitive remediation therapy effects on brain activity and cognition in schizophrenia

TableS1. Comparison of PANSS, cognitive function and skill performance at baseline and post-treatment in CCRT and TAU groups

	CCRT (n=12)		TAU (n=11)		effect of group		effect of time		group*time interaction		Cohen's d
	mean	std	mean	std	F	*P*	F	*P*	F	*P*	
**Symptoms**											
PANSS											
Baseline	65.73	10.44	62.30	8.12	2.32	0.15	0.72	0.41	0.35	0.56	-0.24
Follow-up	62.00	10.46	60.40	5.82							
PANSS positive											
Baseline	13.45	4.46	10.90	3.00	7.48	**0.01**	0.38	0.55	0.36	0.55	0.26
Follow-up	13.27	4.00	9.80	1.87							
PANSS negative											
Baseline	20.18	4.71	20.00	3.94	0.03	0.87	1.98	0.18	0.20	0.66	-0.24
Follow-up	18.36	4.11	18.90	2.73							
PANSS general psychiosis											
Baseline	28.45	3.93	27.70	3.09	0.62	0.44	1.98	0.18	1.29	0.27	-0.42
Follow-up	26.73	4.54	27.60	4.22							
**Cognitive function**											
Trail making test											
Baseline	41.63	10.89	37.48	13.98	1.96	0.18	0.09	0.77	8.14	**0.01**	0.97
Follow-up	45.82	8.10	35.80	10.85							
Spatial span test											
Baseline	47.78	9.72	42.11	4.55	2.46	0.13	2.30	0.15	0.22	0.65	0.16
Follow-up	51.67	10.24	44.68	12.15							
Digit sequencing test											
Baseline	48.73	12.90	42.70	7.74	1.99	0.18	0.02	0.89	0.002	0.96	-0.17
Follow-up	46.28	13.25	41.94	11.08							
Mazes test											
Baseline	39.71	16.30	36.19	11.13	1.29	0.27	0.63	0.44	4.11	**0.06**	0.81
Follow-up	50.57	14.50	39.65	9.78							

Note: PANSS = Positive and Negative Syndrome Scale

Effect of treatment across age, age of illness onset, duration of illness

In this study, we performed correlation analysis to examine relationship between clinical symptoms, cognitive function and brain activity changes with age, age of illness onset, duration of illness. Firstly, we used non-parametric test and found that all the above variables of our data (including clinical symptoms change, cognitive function and brain activity changes, age, age of illness onset, duration of illness) had normal distribution. Then Pearson correlation analysis was performed to explore relationship. The results showed that brain changes in Brainstem had significant negative correlation with onset age ($r=-0.471$, $P=0.042$). We have also added this in the limitation.
